# Supplementary material for: Environmental Enrichment Prevents Gut Dysbiosis Progression and Enhances Glucose Metabolism in High-Fat Diet-Induced Obese Mice
Source: Int J Mol Sci. 2024 Jun 24;25(13):6904. doi: 10.3390/ijms25136904 (PMC11241766; doi:10.3390/ijms25136904)
Supplement: Supplementary file 1 [file ijms-25-06904-s001.zip › Manzo et al Supplementary Figure S1.pdf]

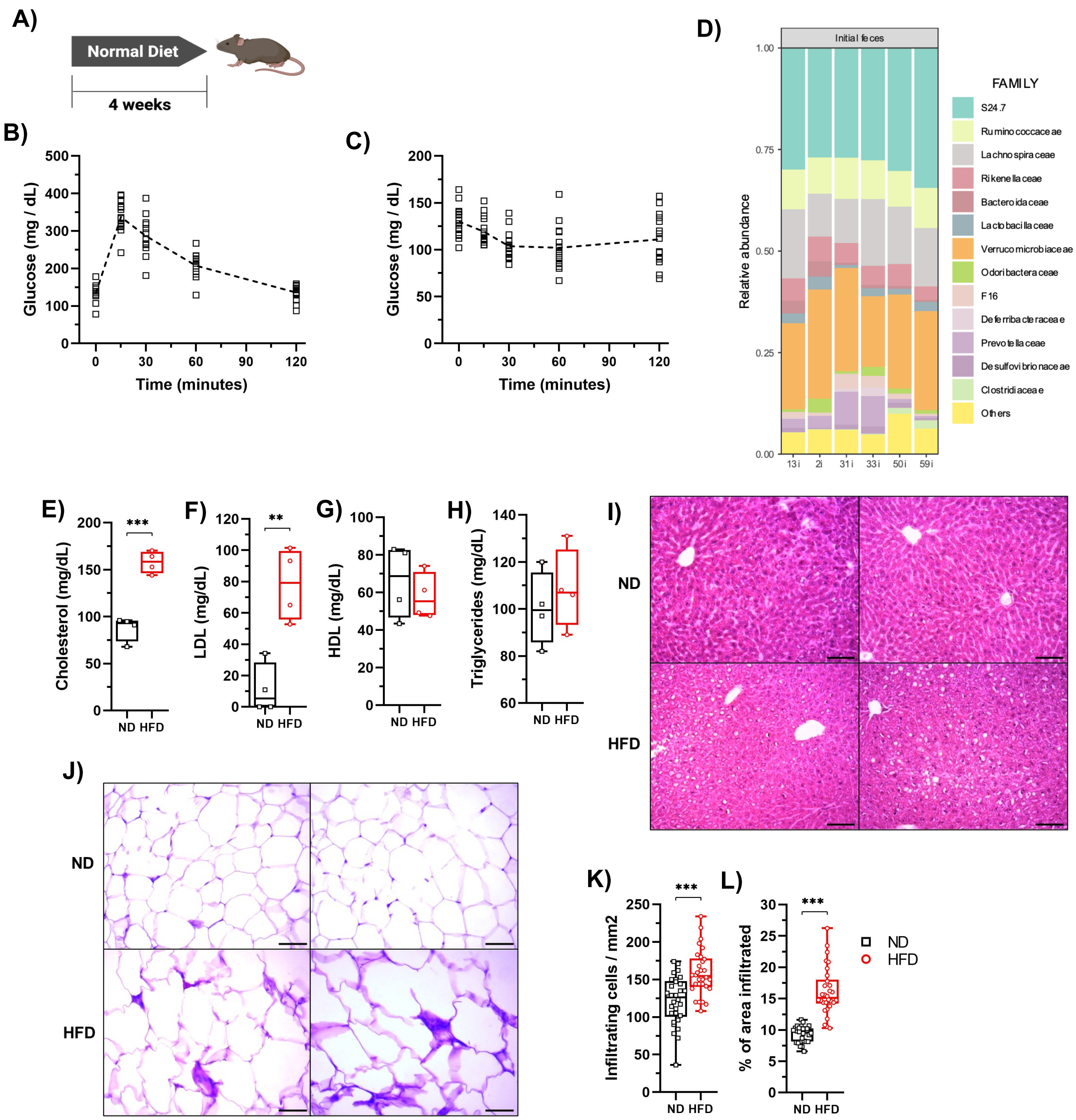

Supplementary figure S1. **High Fat Diet feeding induces metabolic alterations in mice**. A) C57BL/6 mice (3-4 weeks old) were fed with a regular chow diet (ND) for four weeks while gut microbiota was homogenized to eliminate compositional differences. Animals were starved for 7 hours, and glucose tolerance (B) and insulin resistance (C) tests were performed. After four weeks, microbiota homogenization among individual mice was confirmed by 16S rRNA sequencing. D) Microbiota composition expressed as the relative abundance of the main families (n=6). After microbiota homogenization, mice were fed with either normal diet (ND) (n=30) or high fat diet (HFD) (n=30) for 12 weeks in standard housing conditions. E) cholesterol, F) LDL, G) HDL and H) triglyceride serum levels (n=4). I) Representative photos of liver hematoxylin-eosin staining. Scale bars: 100 µm. J) Representative photos of hematoxylin-eosin staining of epididymal white adipose tissue. Scale bar: 100 µm. K) Percentage of infiltrating cell count and L) infiltrated area in white adipose. Immune cell infiltration was calculated from 3 mice from each group in 10 different fields (20x). Bars represent the mean ± SEM. **P < 0.01, ***P<0.001 vs. ND group [Unpaired t-test (E-I, K and L)].
