# Supplementary material for: Environmental Enrichment Prevents Gut Dysbiosis Progression and Enhances Glucose Metabolism in High-Fat Diet-Induced Obese Mice
Source: Int J Mol Sci. 2024 Jun 24;25(13):6904. doi: 10.3390/ijms25136904 (PMC11241766; doi:10.3390/ijms25136904)
Supplement: Supplementary file 1 [file ijms-25-06904-s001.zip › Manzo et al Supplementary Figure S2.pdf]

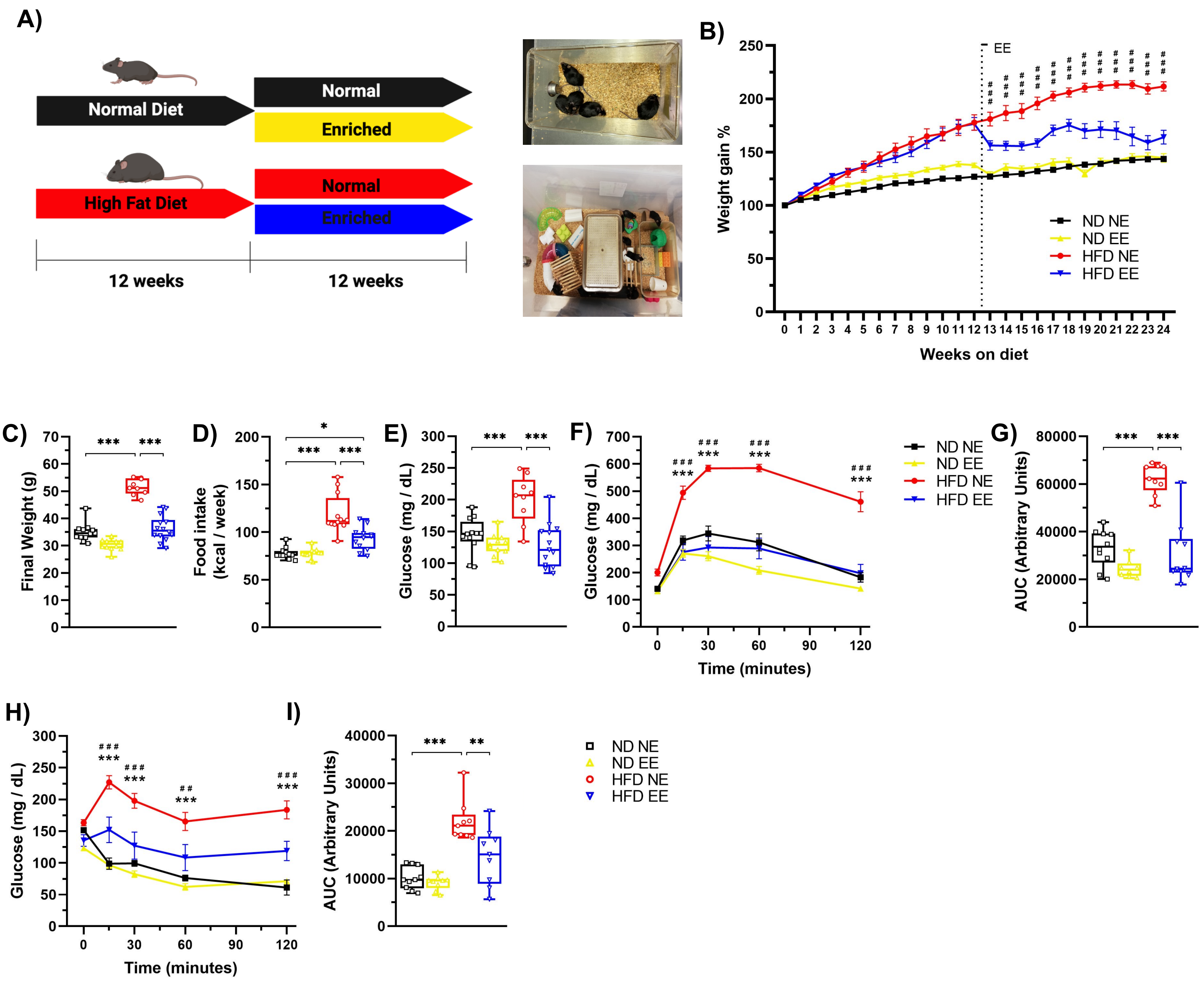

Supplementary figure S2. **Environmental enrichment reduces the metabolic alterations caused by a high fat diet consumption**. A) Experimental design: C57BL/6N mice in standard housing conditions were fed with regular chow diet (ND) or high fat diet (HFD) for 12 weeks and then were separated into standard housing conditions (NE) or environmental enrichment (EE) for an additional 12 weeks. Mice were fed with the same diet they had before they were separated into different housing conditions. Representative photo of a standard housing and EE cage is shown. Experimental groups: ND NE, ND EE, HFD NE, HFD EE. At week 26 mice were euthanized. B) Average weekly weight along the entire experiment. C) Final weight at week 12 for the HFD group (n=30); or at week 25 for the HFD NE (n=10), and HFD EE (n=15) groups. D) Weekly average of food intake for the HFD (n=30), HFD NE (n=10), and HFD EE (n=15) groups. E) At week 12, mice were fasted for 6 h to measure blood glucose levels (n=20). Then, glucose tolerance test (GTT) and insulin tolerance test (ITT) were determined. F) GTT (n=10). G) Area under the curve (AUC) for the GTT (n=10). (H) ITT (n=10). I) AUC for the ITT (n=10). Bars represent the mean ± SEM. *P < 0.05, **P < 0.01, ***P<0.001 vs. ND NE mice # vs. HFD EE [two-way ANOVA followed by a Bonferroni post-hoc test (B-M)].
