# Supplementary material for: Environmental Enrichment Prevents Gut Dysbiosis Progression and Enhances Glucose Metabolism in High-Fat Diet-Induced Obese Mice
Source: Int J Mol Sci. 2024 Jun 24;25(13):6904. doi: 10.3390/ijms25136904 (PMC11241766; doi:10.3390/ijms25136904)
Supplement: Supplementary file 1 [file ijms-25-06904-s001.zip › Manzo et al Supplementary Figure S3.pdf]

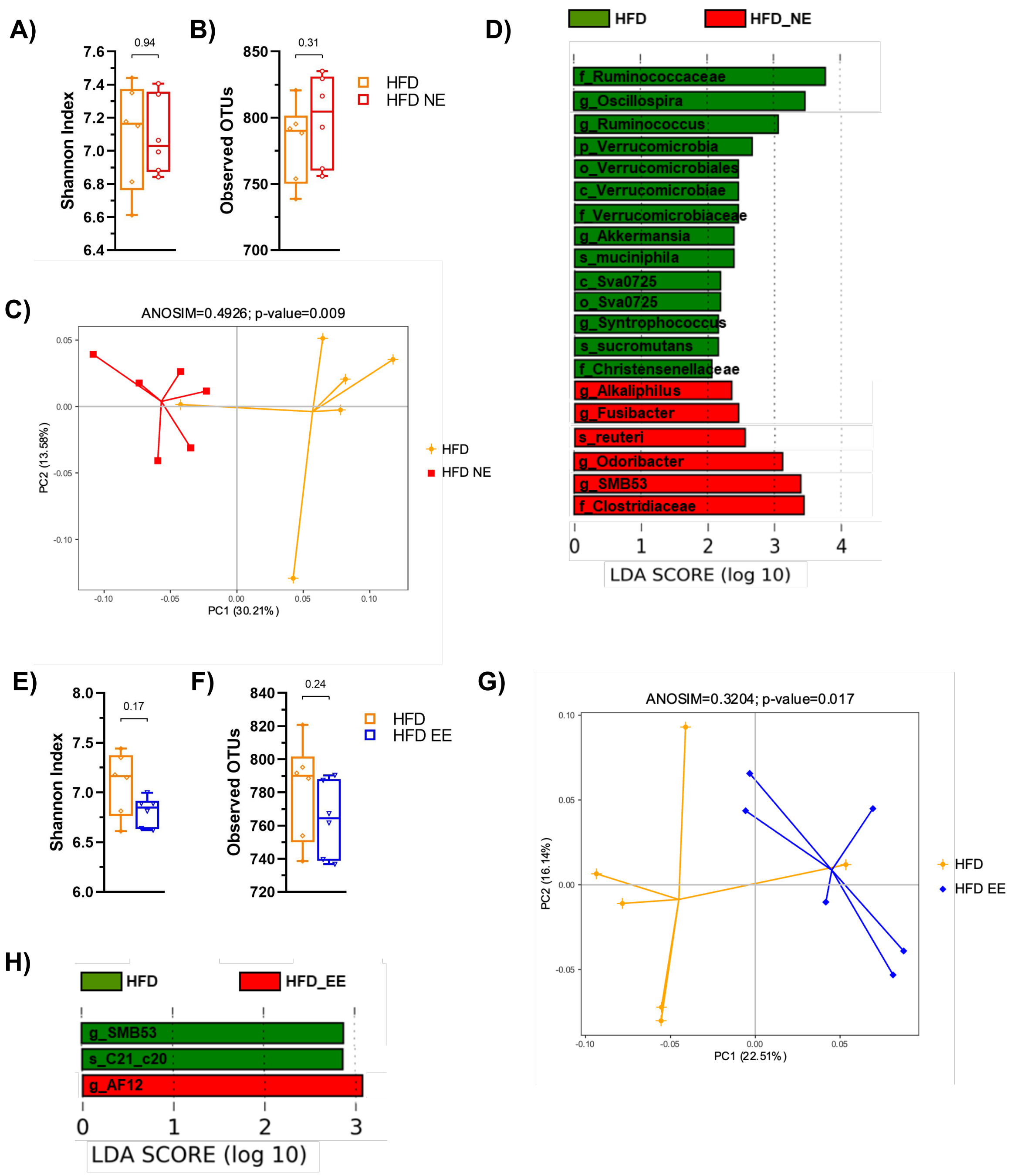

Supplementary figure S3. **Environmental enrichment arrests the progression of gut microbiota dysbiosis induced by long term high fat diet consumption**. Gut microbiota from C57BL/6N mice fed with HFD for 12 weeks in standard housing conditions (HFD) or maintained for an additional 12 weeks in standard housing (HFD NE) conditions was subjected to high-throughput sequencing targeting the V3 and V4 regions of the 16S rRNA gene. Alpha diversity evaluation of gut microbiota richness and diversity by measuring the Shannon index (A) and operational taxonomic units (OTUs; B). C) Principal component analysis showing the beta diversity clustering of the gut microbiota from mice fed with an HFD for 12 (HFD) or 24 weeks and maintained in standard housing conditions (HFD NE). D) LEfSe comparing bacterial class, order, family, genus, and specie between the HFD and HFD NE groups. See supplementary table 3 for the taxonomic ranks of each bacterial. C57BL/6N mice were fed with HFD for 12 weeks in standard housing conditions (HFD) or maintained for an additional 12 weeks in environmental enrichment (HFD EE) and their gut microbiota sequenced by targeting the V3 and V4 regions of the 16S rRNA gene. Alpha diversity evaluation of gut microbiota richness and diversity by measuring the Shannon index (E) and operational taxonomic units (OTUs; F). G) Principal component analysis showing the beta diversity clustering of the gut microbiota from mice fed with an HFD for 12 (HFD) or 24 weeks and maintained in EE conditions (HFD NE). H) LEfSe comparing bacterial genus, and specie between the HFD and HFD EE groups. p_: Phylum; c_: Class; o_: Order; f_: Family; g_: Genus; s_: Specie. See supplementary table 4 for the taxonomic ranks of each bacterial.
