# Supplementary material for: Environmental Enrichment Prevents Gut Dysbiosis Progression and Enhances Glucose Metabolism in High-Fat Diet-Induced Obese Mice
Source: Int J Mol Sci. 2024 Jun 24;25(13):6904. doi: 10.3390/ijms25136904 (PMC11241766; doi:10.3390/ijms25136904)
Supplement: Supplementary file 1 [file ijms-25-06904-s001.zip › Manzo et al Supplementary Figure S4.pdf]

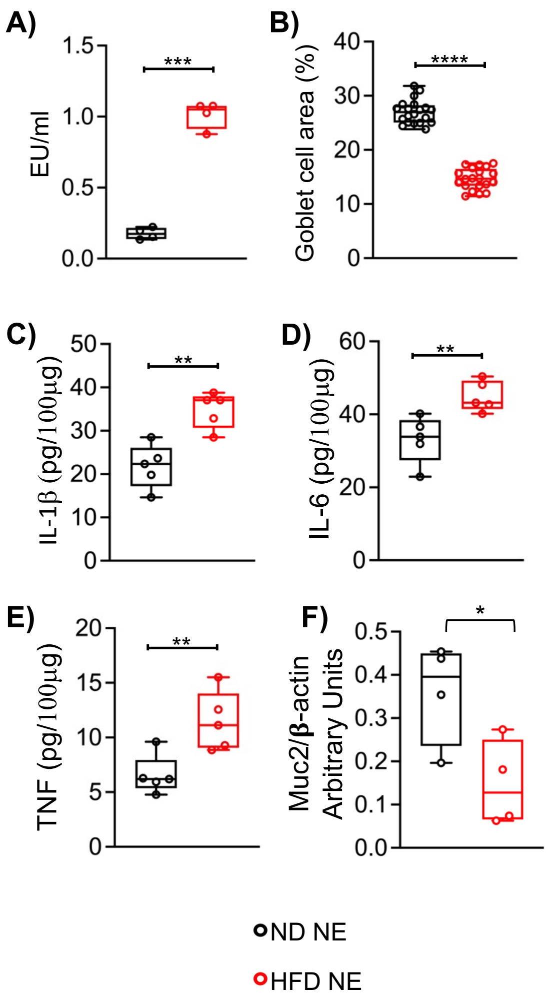

Supplementary figure S4. **High Fat Diet feeding induces an inflammatory response in the proximal colon in mice**. C57BL/6N mice fed with regular chow diet (ND) or high fat diet (HFD) for 24 weeks in standard housing conditions (NE) were euthanized and the proximal colon and serum were collected for further analysis. A) Circulating lipopolysaccharide levels. B) Percentage of goblet cell area. C) IL-1β, D) IL-6 and E) TNF levels in the proximal colon. F) Muc2 protein levels in the proximal colon respect. Data are mean±S.E.M. **P<0.01, ***P<0.001, ***P<0.001, ****P<0.001 versus HFD or HFD NE (unpaired two-tailed t-test).
