# Supplementary material for: Environmental Enrichment Prevents Gut Dysbiosis Progression and Enhances Glucose Metabolism in High-Fat Diet-Induced Obese Mice
Source: Int J Mol Sci. 2024 Jun 24;25(13):6904. doi: 10.3390/ijms25136904 (PMC11241766; doi:10.3390/ijms25136904)
Supplement: Supplementary file 1 [file ijms-25-06904-s001.zip › Manzo et al Supplementary Table S2.pdf]

Table S2. **Taxonomic ranks of each bacterial identified in the HFD NE and HFD EE groups**

TAXA ABBREVIATION	TAXA ID
**o_SM1D11**	k__Bacteria;p__Cyanobacteria;c__4C0d-2;o__SM1D11
**f_Clostridiaceae**	k__Bacteria;p__Firmicutes;c__Clostridia;o__Clostridiales;f__Clostridiaceae
**f_Acidaminobacteraceae**	k__Bacteria;p__Firmicutes;c__Clostridia;o__Clostridiales; f__[Acidaminobacteraceae]
**f_Veillonellaceae**	k__Bacteria;p__Firmicutes;c__Clostridia;o__Clostridiales; f__Veillonellaceae
**f_Peptostreptococcaceae**	k__Bacteria;p__Firmicutes;c__Clostridia;o__Clostridiales; f__Peptostreptococcaceae
**g_SMB53**	k__Bacteria;p__Firmicutes;c__Clostridia;o__Clostridiales; f__Clostridiaceae;g__SMB53
**g_Allobaculum**	k__Bacteria;p__Firmicutes;c__Erysipelotrichi;o__Erysipelotrichales; f__Erysipelotrichaceae;g__Allobaculum
**g_Fusibacter**	k__Bacteria;p__Firmicutes;c__Clostridia;o__Clostridiales; f__[Acidaminobacteraceae];g__Fusibacter
**g_Alkaliphilus**	k__Bacteria;p__Firmicutes;c__Clostridia;o__Clostridiales; f__Clostridiaceae;g__Alkaliphilus
**g_Tepidimicrobium**	k__Bacteria;p__Firmicutes;c__Clostridia;o__Clostridiales; f__[Tissierellaceae];g__Tepidimicrobium
**g_Clostridiisalibacter**	k__Bacteria;p__Firmicutes;c__Clostridia;o__Clostridiales; f__Clostridiaceae;g__Clostridiisalibacter
**s_C21_c20**	k__Bacteria;p__Proteobacteria;c__Deltaproteobacteria;o__Desulfovibrionales; f__Desulfovibrionaceae;g__Desulfovibrio;s__C21_c20
**s_reuteri**	k__Bacteria;p__Firmicutes;c__Bacilli;o__Lactobacillales;f__Lactobacillaceae; g__Lactobacillus;s__reuteri
**c_Sva0725**	k__Bacteria;p__Acidobacteria;c__Sva0725
**o_Sva0725**	k__Bacteria;p__Acidobacteria;c__Sva0725;o__Sva0725
**f_Bacteroidaceae**	k__Bacteria;p__Bacteroidetes;c__Bacteroidia;o__Bacteroidales; f__Bacteroidaceae
**f_Prevotellaceae**	k__Bacteria;p__Bacteroidetes;c__Bacteroidia;o__Bacteroidales; f__Prevotellaceae
**f_Dehalobacteriaceae**	k__Bacteria;p__Firmicutes;c__Clostridia;o__Clostridiales; f__Dehalobacteriaceae
**g_Bacteroides**	k__Bacteria;p__Bacteroidetes;c__Bacteroidia;o__Bacteroidales; f__Bacteroidaceae;g__Bacteroides
**g_Syntrophococcus**	k__Bacteria;p__Firmicutes;c__Clostridia;o__Clostridiales;f__Lachnospiraceae; g__Syntrophococcus
**g_Prevotella**	k__Bacteria;p__Bacteroidetes;c__Bacteroidia;o__Bacteroidales; f__Prevotellaceae;g__Prevotella
**g_Dehalobacterium**	k__Bacteria;p__Firmicutes;c__Clostridia;o__Clostridiales; f__Dehalobacteriaceae;g__Dehalobacterium
**g_Butyricimonas**	k__Bacteria;p__Bacteroidetes;c__Bacteroidia;o__Bacteroidales; f__[Odoribacteraceae];g__Butyricimonas
**s_sucromutans**	k__Bacteria;p__Firmicutes;c__Clostridia;o__Clostridiales;f__Lachnospiraceae; g__Syntrophococcus;s__sucromutans

c_: Class; o_: Order; f_: Family; g_: Genus; s_: Specie.
Experimental groups: mice fed with a high fat diet for 24 weeks and maintained in standard housing (HFD NE) or in environmental enrichment (HFD EE) conditions.
