# Supplementary material for: Environmental Enrichment Prevents Gut Dysbiosis Progression and Enhances Glucose Metabolism in High-Fat Diet-Induced Obese Mice
Source: Int J Mol Sci. 2024 Jun 24;25(13):6904. doi: 10.3390/ijms25136904 (PMC11241766; doi:10.3390/ijms25136904)
Supplement: Supplementary file 1 [file ijms-25-06904-s001.zip › Manzo et al Supplementary Table S3.pdf]

Table S3. **Taxonomic ranks of each bacterial identified in the HFD and HFD NE groups**

TAXA ABBREVIATION	TAXA ID
**f_Ruminococcaceae**	k__Bacteria;p__Firmicutes;c__Clostridia;o__Clostridiales;f__Ruminococcaceae
**g_Oscillospira**	k__Bacteria;p__Firmicutes;c__Clostridia;o__Clostridiales;f__Ruminococcaceae; g__Oscillospira
**g_Ruminococcus**	k__Bacteria;p__Firmicutes;c__Clostridia;o__Clostridiales;f__Ruminococcaceae; g__Ruminococcus
**p_Verrucomicrobia**	k__Bacteria;p__Verrucomicrobia
**o_Verrucomicrobiales**	k__Bacteria;p__Verrucomicrobia;c__Verrucomicrobiae;o__Verrucomicrobiales
**c_Verrucomicrobiae**	k__Bacteria;p__Verrucomicrobia;c__Verrucomicrobiae
**f_Verrucomicrobiaceae**	k__Bacteria;p__Verrucomicrobia;c__Verrucomicrobiae;o__Verrucomicrobiales; f__Verrucomicrobiaceae
**g_Akkermansia**	k__Bacteria;p__Verrucomicrobia;c__Verrucomicrobiae;o__Verrucomicrobiales; f__Verrucomicrobiaceae;g__Akkermansia
**s_muciniphila**	k__Bacteria;p__Verrucomicrobia;c__Verrucomicrobiae;o__Verrucomicrobiales; f__Verrucomicrobiaceae;g__Akkermansia;s__muciniphila
**c_Sva0725**	k__Bacteria;p__Acidobacteria;c__Sva0725
**o_Sva0725**	k__Bacteria;p__Acidobacteria;c__Sva0725;o__Sva0725
**g_Syntrophococcus**	k__Bacteria;p__Firmicutes;c__Clostridia;o__Clostridiales;f__Lachnospiraceae;g__ Syntrophococcus
**s_sucromutans**	k__Bacteria;p__Firmicutes;c__Clostridia;o__Clostridiales;f__Lachnospiraceae;g__ Syntrophococcus;s__sucromutans
**f_Christensenellaceae**	k__Bacteria;p__Firmicutes;c__Clostridia;o__Clostridiales;f__Christensenellaceae
**g_Alkaliphilus**	k__Bacteria;p__Firmicutes;c__Clostridia;o__Clostridiales; f__Clostridiaceae;g__Alkaliphilus
**g_Fusibacter**	k__Bacteria;p__Firmicutes;c__Clostridia;o__Clostridiales; f__[Acidaminobacteraceae];g__Fusibacter
**s_reuteri**	k__Bacteria;p__Firmicutes;c__Bacilli;o__Lactobacillales;f__Lactobacillaceae; g__Lactobacillus;s__reuteri
**g_Odoribacter**	k__Bacteria;p__Bacteroidetes;c__Bacteroidia;o__Bacteroidales; f__[Odoribacteraceae];g__Odoribacter
**g_SMB53**	k__Bacteria;p__Firmicutes;c__Clostridia;o__Clostridiales; f__Clostridiaceae;g__SMB53
**f_Clostridiaceae**	k__Bacteria;p__Firmicutes;c__Clostridia;o__Clostridiales;f__Clostridiaceae

p_: Phylum; c_: Class; o_Order; f_:Family; g_Genus; s_Specie
Experimental groups: mice fed with a high fat diet for 12 weeks in standard housing conditions (HFD); mice fed for 24 weeks with a high fat diet and maintained in standard housing conditions (HFD NE).
