# Supplementary material for: Environmental Enrichment Prevents Gut Dysbiosis Progression and Enhances Glucose Metabolism in High-Fat Diet-Induced Obese Mice
Source: Int J Mol Sci. 2024 Jun 24;25(13):6904. doi: 10.3390/ijms25136904 (PMC11241766; doi:10.3390/ijms25136904)
Supplement: Supplementary file 1 [file ijms-25-06904-s001.zip › Manzo et al Supplementary Table S4.pdf]

Table S4. **Taxonomic ranks of each bacterial identified in the HFD and HFD EE groups**

TAXA ABBREVIATION	TAXA ID
**g_SMB53**	k__Bacteria;p__Firmicutes;c__Clostridia;o__Clostridiales; f__Clostridiaceae;g__SMB53
**s_C21_c20**	k__Bacteria;p__Proteobacteria;c__Deltaproteobacteria;o__Desulfovibrionales; f__Desulfovibrionaceae;g__Desulfovibrio;s__C21_c20
**g_AF12**	k__Bacteria;p__Bacteroidetes;c__Bacteroidia;o__Bacteroidales;f__Rikenellaceae;g__AF12

p_: Phylum; c_: Class; o_: Order; f_:Family; g_: Genus; s_: Specie
Experimental groups: mice fed with a high fat diet for 12 weeks in standard housing conditions (HFD); mice fed for 24 weeks with a high fat diet and maintained in environmental enrichment conditions (HFD EE).
