# Supplementary figures and images for: Environmental Enrichment Prevents Gut Dysbiosis Progression and Enhances Glucose Metabolism in High-Fat Diet-Induced Obese Mice
Source: Int J Mol Sci. 2024 Jun 24;25(13):6904. doi: 10.3390/ijms25136904 (PMC11241766; doi:10.3390/ijms25136904)

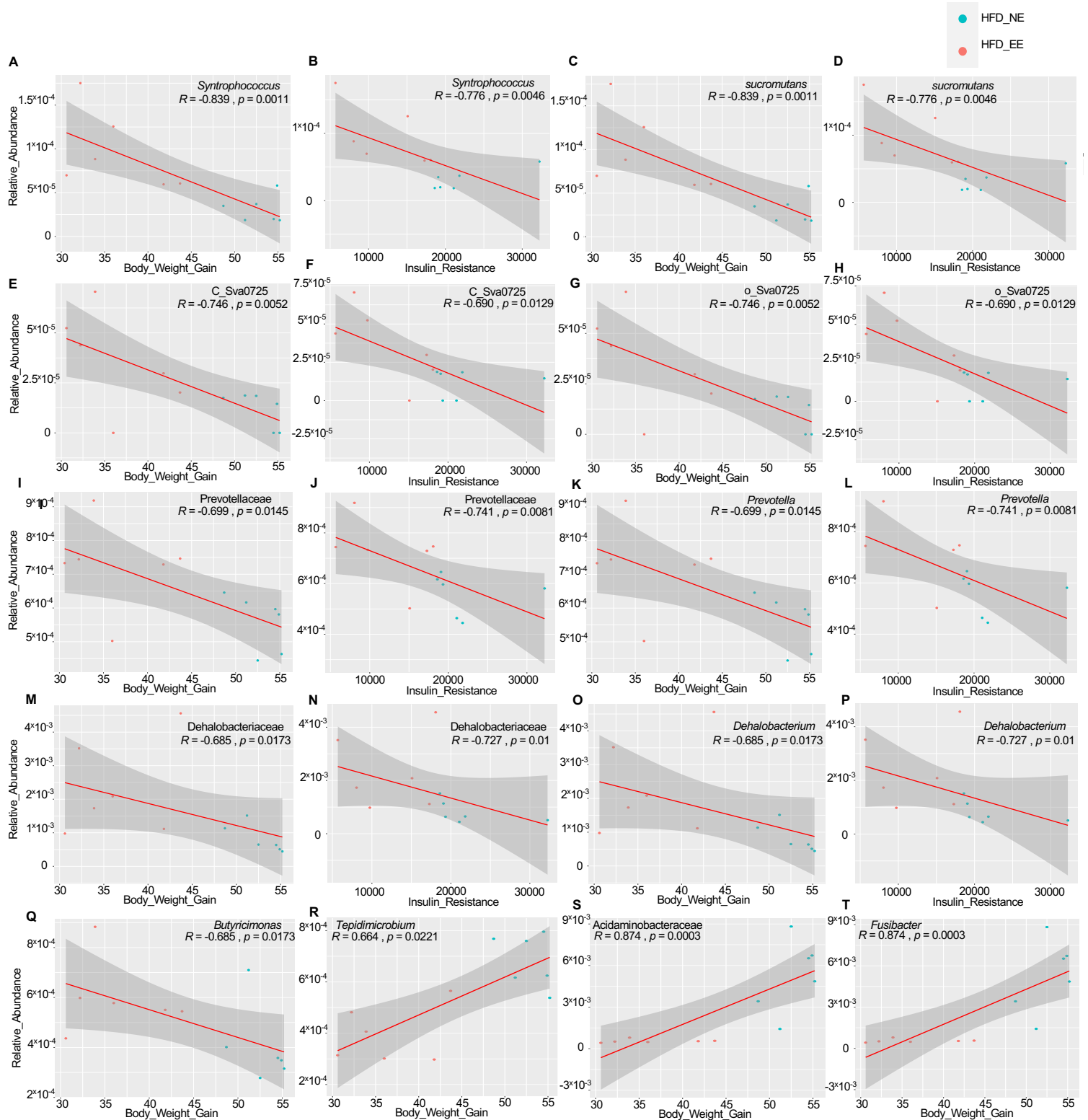

Supplement: Supplementary file 1 [file ijms-25-06904-s001.zip › Manzo et al Supplementary Figure S5.pdf]
